# Regional Homogeneity of Resting-State Brain Activity Suppresses the Effect of Dopamine-Related Genes on Sensory Processing Sensitivity

**DOI:** 10.1371/journal.pone.0133143

**Published:** 2015-08-26

**Authors:** Chunhui Chen, Daiming Xiu, Chuansheng Chen, Robert Moyzis, Mingrui Xia, Yong He, Gui Xue, Jin Li, Qinghua He, Xuemei Lei, Yunxin Wang, Bin Liu, Wen Chen, Bi Zhu, Qi Dong

**Affiliations:** 1 State Key Laboratory of Cognitive Neuroscience and Learning and IDG/McGovern Institute for Brain Research, Beijing Normal University, Beijing, 100875,China; 2 Center for Collaboration and Innovation in Brain and Learning Sciences, Beijing Normal University, Beijing, 100875, China; 3 Department of Psychology and Social Behavior, University of California, Irvine, California, 92697, United States of America; 4 Department of Biological Chemistry and Institute of Genomics and Bioinformatics, University of California, Irvine, California, 92697, United States of America; 5 Faculty of Psychology, Southwest University, Beibei, Chongqing, 400715, China; 6 Institute of Psychology, Division of Psychopathology and Clinic Intervention, University of Zurich, Zurich, CH-8050, Switzerland; Institute of Psychology, Chinese Academy of Sciences, CHINA

## Abstract

Sensory processing sensitivity (SPS) is an intrinsic personality trait whose genetic and neural bases have recently been studied. The current study used a neural mediation model to explore whether resting-state brain functions mediated the effects of dopamine-related genes on SPS. 298 healthy Chinese college students (96 males, mean age = 20.42 years, SD = 0.89) were scanned with magnetic resonance imaging during resting state, genotyped for 98 loci within the dopamine system, and administered the *Highly Sensitive Person Scale*. We extracted a “gene score” that summarized the genetic variations representing the 10 loci that were significantly linked to SPS, and then used path analysis to search for brain regions whose resting-state data would help explain the gene-behavior association. Mediation analysis revealed that temporal homogeneity of regional spontaneous activity (ReHo) in the precuneus actually suppressed the effect of dopamine-related genes on SPS. The path model explained 16% of the variance of SPS. This study represents the first attempt at using a multi-gene voxel-based neural mediation model to explore the complex relations among genes, brain, and personality.

## Introduction

The search for the biological basis of personality traits has been facilitated by the recent developments in both molecular genetics [1] and brain imaging techniques [2, 3]. Thus far, however, the two approaches have rarely been integrated. Few studies have treated brain functions as the intermediate phenotype between genes and personality although about a decade ago it had been proposed that brain can be an intermediate phenotype between genes and behavior [4]. The current study used the brain-mediation approach to investigate whether resting-state brain activities mediated the previously documented effects [5] of dopamine-related genes on the personality trait of sensory processing sensitivity (SPS).

SPS is a basic personality trait characterized by sensitivity to environmental and social stimuli [6, 7]. According to Aron, et al. [7], unlike other individual personality traits, sensitivity is a “meta-personality” trait observed in more than 100 species and is of vital importance to their survival. In terms of genetic correlates, Chen et al. [5] found that the cumulative effects of dopamine-related genes accounted for 12% of the variance of SPS. Specifically, 10 SNPs of 7 dopamine-related genes made independent contributions to SPS. To examine the mediating role of brain functions in the above genetic effect, we focused on resting-state brain activity because it reflects the brain’s intrinsic functional architecture [8], is modulated by dopamine [9–11], and has been associated with personality [12, 13]. In terms of relevant brain regions, previous research has implicated the orbitofrontal cortex, precuneus, middle temporal gyrus, cingulate, and insula in personality traits in general and SPS in particular [14–16]. Therefore, we hypothesized that resting-state brain activity in the above regions would mediate the effect of dopamine-related genes on SPS.

Thus far, at least five studies have successfully examined brain-mediated genetic effects on behavioral outcomes such as superiority illusion, harm avoidance, anxiety traits, and cognitive control [11, 17–20]. Unlike previous research that used a single genetic locus, however, we used a “gene score” [21, 22] to better represent genetic variations of the whole dopamine system and to link them to the complex and necessarily polygenic trait of SPS [23, 24]. In addition, our study included a larger sample size (298 healthy Chinese adults) than previous studies in this area which ranged from 25 to 160 subjects.

## Materials and Methods

### Participants

Of the original 480 subjects in Chen et al.[5], 298 well called back to get resting-state data collected (96 males, mean age = 20.42 years, SD = 0.89). All participants were Han Chinese undergraduate students recruited from Beijing Normal University. They had no history of neurological or psychiatric disorders according to self-report and Magnetic Resonance Imaging (MRI) screening questionnaires. None of them met the criterion for major depression according to the Beck Depression Inventory [25] or for alcohol or nicotine dependence according to the Alcohol Use Disorders Identification Test [26] and the Fagerström Test for Nicotine Dependence [27].Written informed consent was obtained from each participant. This experiment was approved by the IRB of the State Key Laboratory of Cognitive Neuroscience and Learning at Beijing Normal University, China.

### Personality Assessment

*The Highly Sensitive Person Scale* [6] was used to measure participants’ SPS. This scale measured a single construct that had small to moderate correlations with but separated from introversion, neuroticism and emotional reactions [7]. It includes 27 questions about sensitivity, involving having a rich and complex inner life, and being conscientious and deeply moved by the arts and music, to being more shaken than others by changes in one’s life, having more difficulty performing a task when being observed, startling easily, and being more sensitive to pain, hunger, and caffeine. For example, ‘‘Are you easily overwhelmed by strong sensory input?”, ‘‘Do other people’s moods affect you?” and ‘‘Do you tend to be very sensitive to pain?” Participants rated each item on a 7-point scale, 1 = ‘‘Not at all” to 7 = ‘‘Extremely”. The total score of all items was used for analysis. The scale was translated from English to Chinese and back translated and verified through a bilingual group discussion, and then pilot tested. The resulting Chinese version had high internal consistency (*α* = 0.82).

### Gene Score Calculation

**Fig 1:**
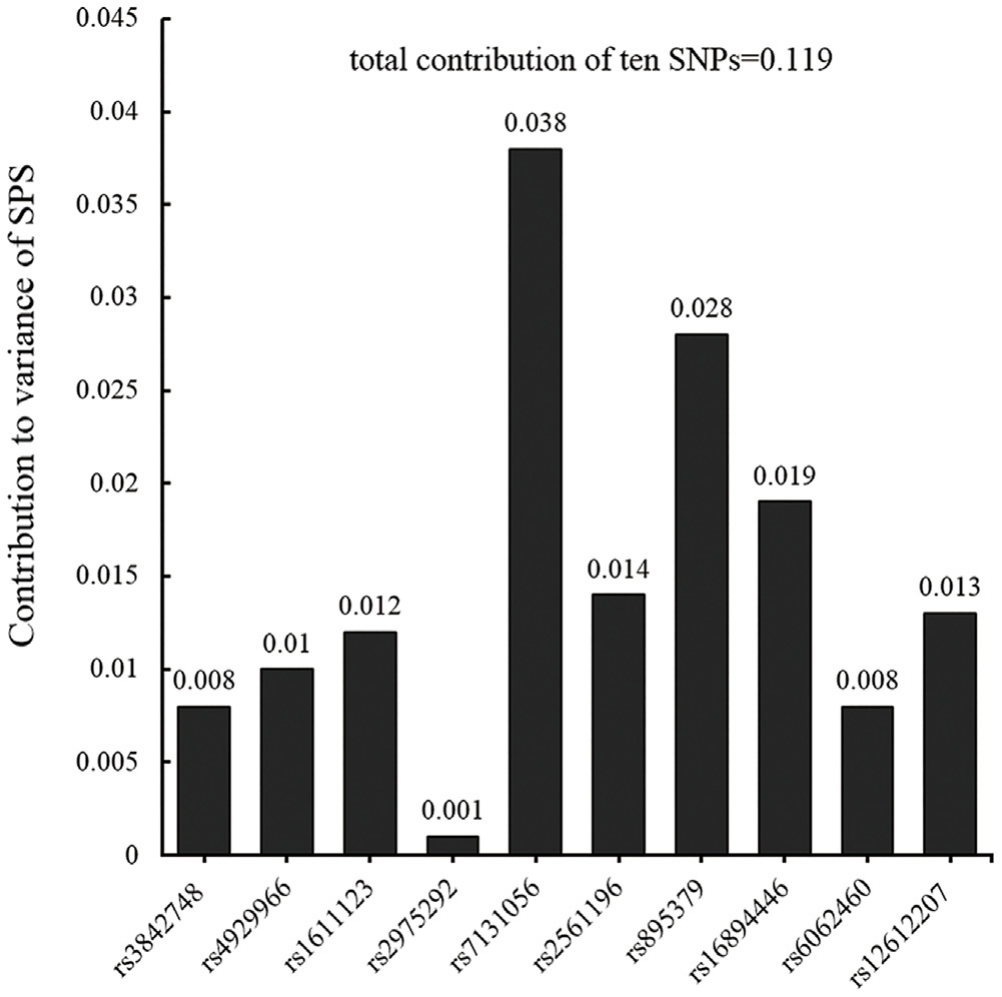
Contributions of identified dopamine-related SNPs to the variance of SPS. Each SNP was individually regressed to the score of SPS; for total contribution, all ten SNPs were simultaneously included in the regression analysis.

In our previous study [5], we firstly selected 16 genes in dopamine system, then preprocessed the gene data (cleaning the low-frequency and Hardy-Weinberg disequilibrium alleles, excluding high LD SNPs), and finally acquired 98 representative polymorphisms (details of all SNPs genotyped can be found in supplementary S1 Table of Chen et al.[5]). Through a multi-step approach (ANOVA followed by multiple regression and permutation), of these polymorphisms, 10 SNPs were identified to be associated with sensitivity and their total contributions were estimated using multiple regression. They were rs3842748 and rs4929966 of *TH*; rs1611123 of *DβH*; rs2975292 of *SLC6A3*; rs7131056 of *DRD2;* rs2561196, rs895379 and rs16894446 of *NLN*; rs6062460 of *NTSR1*; and rs12612207 of *NTSR2*. Contributions of each identified SNP and their total effect are shown in Fig 1. See Chen et al.[5] for details of locus selection, genotyping, quality control, and analysis. Following de Quervain and Papassotiropoulos [21], we created a single gene score to represent the 10 polymorphic loci by coding genotypes as 1 for major homozygotes, 2 for heterozygotes, and 3 for minor homozygotes (the genotype coding in the regression model); multiplying the codes with their effect sizes (the coefficients in the regression model, can be positive or negative, see Chen, Chen. 2011); and summing up the weighted effects across all ten polymorphisms. This gene score was positively correlated with SPS (*r* = 0.36, *p* < 0.01): Subjects with higher gene scores were more sensitive.

### Image Acquisition and Analysis

Magnetic resonance images were collected using a 3-Tesla Siemens Trio system in the Brain Imaging Center of Beijing Normal University. Participants lay supine with head snugly secured by a band and foam pads to minimize head motion. Each participant underwent an eight-minute resting-state functional MRI (RS-fMRI) scanning session and a 3D anatomic session. During the RS-fMRI session, the participants were instructed to close their eyes and keep still and relax, without thinking about anything in particular. Resting-state images were obtained with the following parameters: 33 axial slices, thickness/gap = 3/0.6 mm, in-plane resolution = 64×64, repetition time (TR) = 2000 ms, echo time (TE) = 30 ms, flip angle = 90°, field of view (FOV) = 200×200 mm^2^. The 3D T1-weighted magnetization-prepared rapid gradient echo (MPRAGE) image was acquired with the following parameters: 128 sagital slices, slice thickness/gap = 1.33/0 mm, in-plane resolution = 256×256, TR = 2530 ms, TE = 3.39 ms, inversion time (Ti) = 1100 ms, flip angle = 7°, FOV = 256×256 mm^2^.

Data Processing Assistant for Resting-State fMRI (DPARSF), Resting-State fMRI Data Analysis Toolkit (REST) [28] (http://www.restfmri.net), and Statistical Parametric Mapping (SPM8, www.fil.ion.ucl.ac.uk/spm) were used to analyze the RS-fMRI data. Steps included: (1) discarding the first 10 volumes to allow participants to get used to the fMRI scanning environment; (2) correcting for within-scan acquisition time differences between slices and head motions (no participant had head motion more than 2.0 mm of displacement or 2.0° of rotation throughout the course of the scan); (3) coregistering the T1 image to the mean functional image using a linear transformation; (4) segmenting the coregistered T1 images into grey matter, white matter and cerebrospinal fluid; (5) normalizing the head-motion-corrected functional images to a standard template using the transformation matrix estimated from T1 segmentation and reslicing them to 3 mm isotropic resolution; (6) linear detrending and temporal band-pass filtering (0.01~0.08 Hz); (7) regressing out nuisance signals including the six head motion profiles, global mean signal, cerebrospinal fluid signal, and white matter signal.

Regional homogeneity (ReHo) that reflects the temporal homogeneity of regional spontaneous activity was calculated using Kendall coefficient of concordance based on 27 nearest neighboring voxels [29], then demeaned and smoothed with FWHM of 4mm. We used ReHo because it has been associated with normal variations in functions [30, 31] as well as dysfunctions related to various diseases [32–34]. More relevantly, it has been shown to be sensitive to oral administration of levodopa (a precursor of dopamine) [35] and its individual differences have been associated with genetic variations [36].

To clarify potential artificial effects resulted from head motion, we calculated three head motion indices for each subject: (1) square root of sum of squared movement and (2) square root of sum of squared rotation in three dimension as calculated in Zuo et al.[37], and (3) framewise displacement as calculated in Power et al.[38]. We correlated these three indices with both the gene score and SPS. None of the correlations were significant (*r* ranged from -0.002 to -0.099, *p* from 0.968 to 0.089).

### Mediation Analysis

Mediation models were tested at the voxel and ROI (region of interest) levels. The gene score was used as the independent variable, SPS as the dependent variable, and gender as a control variable; and ReHo of each voxel was used as the mediator for the voxel-level model and the average ReHo indices of the regions of interest were used as the mediators for the ROI-based model. The voxel-level model was run using in-house Matlab codes, and the ROI-based model was run through AMOS (Data for mediation analysis are shown in S1 Table).

### Voxel-based Mediation Testing

The procedure of mediation testing used in the present study was adopted from Wen et al.[39]. There were three steps: (1) to test if the independent variable (gene score) contributed significantly to the dependent variable (SPS); (2) to test if the independent variable contributed significantly to the mediator (ReHo values); and (3) to test if the mediator contributed significantly to the dependent variable controlling for the independent variable. In all steps, gender was included as a control variable. If all three steps showed significant effects (*p* < 0.05), a significant mediation effect was revealed. This procedure was run on each voxel within gray matter and thus resulted in a “mediation brain” map. We tried two ways for multiple comparison correction. One was AFNI 3dFWHMx and 3dClustSim using the following parameters: voxels in mask were 50296 because only grey matter was analyzed, Gaussian filter width (FWHM) was 8.18566×8.15281×7.92518 mm^3^ estimated by 3dFWHMx,individual voxel threshold probability be 0.000125 (0.05×0.05×0.05 for a significant mediator voxel because of three steps in mediation analysis with each using the threshold of *p* < 0.05). The above procedure resulted in a threshold of cluster size > 216 mm^3^ (8 voxels) to be considered significant, corresponding to corrected *p* < 0.05. The second way is using permutation. We generated a gene score by randomly select 10 SNPs, and run the mediation analysis as did with the actual gene score and record the maximum cluster got. We repeated the procedure 1000 times to get a distribution of maximum cluster got from random gene score, and found that the 95% threshold be 12 voxels. To get a more robust result, we used the stricter threshold, only ReHo of clusters larger than 12 voxels were extracted and averaged for the following ROI-based mediation model estimate.

### ROI-based Mediation Model

Mean ReHo of significant clusters was extracted and a mediation model was confirmed with AMOS. We used bootstrap to estimate 95% confidence interval. Gender was included as a covariate. We further confirmed this mediation effect with the Leave-One-Out procedure: we conducted the analysis by excluding data from one subject out and building the mediation model with the remaining subjects, and then used the model to predict the HSP score of the excluded subject. We repeated this procedure for every subject and correlated the predicted HSP scores to the observed ones.

## Results

### Voxel-based Mediation

The mean total score of SPS based on the *Highly Sensitive Person Scale* was 122.40, SD = 15.77. There was no significant gender difference: mean = 120.25, SD = 15.97 for males, and mean = 123.49, SD = 15.61 for females; *t* = 2.88, *p* = 0.09. Scores of both genders were normally distributed: skewness = 0.25, kurtosis = 0.49, *Kolmogorov-Smirnov* test Z = 0.57, *p* = 0.91 for males; skewness = 0.17, kurtosis = 0.34, *Kolmogorov-Smirnov* test Z = 0.76, *p* = 0.61 for females.

After correction of AFNI 3dFWHMx and 3dClustSim, significant mediation effects were found in the precuneus (Pcc; peak MNI coordinate: x = 3, y = -60, z = 33; voxel size = 16 [432 mm^3^]; peak mediating effect = -0.0722) and left inferior temporal gyrus (lITG; peak MNI coordinate: x = -51, y = -21, z = -33; voxel size = 9 [243 mm^3^]; peak mediating effect = 0.0661). However, only the Pcc survived after permutation and was included in the ROI-based analysis.

### ROI-based Mediation

**Fig 2:**
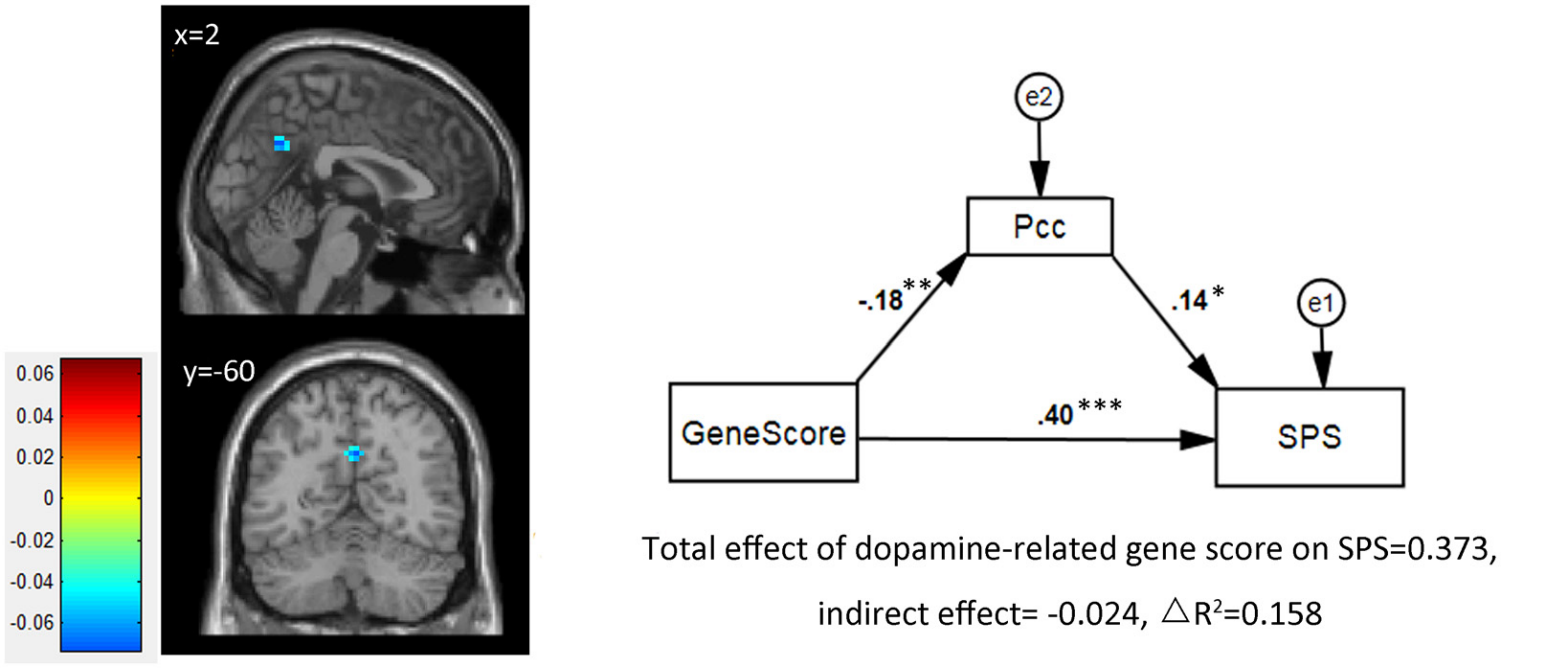
Mediation analysis results. Left: voxel based analysis find a cluster of Precuneus showed significant mediation effect after multiple comparison correction. Right: ROI based analysis confirmed its partial mediation effect. Path coefficients in the graph are standardized regression weights.* *p*<0.05, **p<0.01, ***p<0.001.

We extracted the mean score of Pcc and constructed a mediation model. Results showed a negative prediction from the gene score to ReHo of the Pcc (Pearson correlation: *r* = -.176, *p* = .002; regression weight = -.176, *p* = .002) and a positive prediction from the ReHo of the Pcc to SPS (regression weight = 0.139, *p* = .01) (see Fig 2). These results indicated that higher gene scores of dopamine-related genes led to weaker homogeneity of regional spontaneous activity in the Pcc, which in turn led to higher sensitivity. This pattern of results indicated that instead of traditional mediation, ReHo of the Pcc was a suppressor of the association between dopamine-related genes and SPS [30], with a suppression effect of -6.53% of the total effect. Altogether, the multi-gene-brain mediating model accounted for 15.8% of the variance of SPS (Fig 2).

The Leave-One-Out procedure analysis showed that the correlation between predicted and observed HSP scores was 0.366 (*p = 7*.*5781e-11*), suggesting that the mediation/suppressor model was reliable.

## Discussion

The present study aimed to identify neural mediators between the dopamine-related genetic system and the personality trait of sensory processing sensitivity (SPS). Results showed that, at the voxel level, the precuneus turned out to have a significant suppressor effect instead of a traditional mediation effect. After considering the suppressor effect, the path model accounted for 16% of the variance of SPS. This work contributes to the literature both theoretically and methodologically.

Three previous task-related fMRI studies have suggested that sensitivity to subtle stimuli is related to specific areas in the frontal, parietal and temporal regions, as well as the insula, that subserve working memory, attention, awareness, integration of sensory information, and action planning [14–16]. We found that the Pcc and lITP mediated the associations between dopamine-related genes and SPS. In dorsal pathway, the precuneus has been found to be involved in regulation and integration of higher-order information involving visuo-spatial imagery, episodic memory, and emotional stimuli, especially when self-related mental representations and self-processing are involved [40–42]. Low-frequency oscillations in the resting activity of the precuneus were correlated positively with extraversion and negatively with neuroticism [43, 44]. Of the ventral pathway, the inferior temporal gyrus identified in this study has been found to be related to visual and language learning as well as memory and imagery [45].

These regions are within the dopaminergic pathways, which connect the ventral tegmentum to the cerebral cortex [46]. Dopamine receptors have also been found in temporal cortex [47, 48], the precuneus and its adjacent area [49]. Dopamine acts to modulate the neural activity in the precuneus during visuospatial attention [50] and the functional connectivity between the precuneus and the amygdala during aversive conditioning [51]. Recent studies have also suggested that dopamine can modulate resting-state brain activity and related behaviors. For example, several pharmacological fMRI studies have demonstrated that DA agonist and antagonist drugs modulate resting-state functional connectivity (RSFC) [9, 10, 52], which was further linked to impulsivity [52]. Gordon et al.[53] found that the *DAT1 (SLC6A3)* gene was associated with striato-frontal RSFC, which was in turn related to working memory. Yamada et al.[11] found that the availability of striatal D2 receptor contributed to RSFC, which was linked to one’s illusory superiority over others. Taken together, these results suggested that dopamine-related genes may influence intrinsic activity of the above-mentioned brain regions and consequently SPS. Interestingly, our results showed a suppressor effect. That is, the effect of dopamine-related genes was linked to reduced homogeneity of regional spontaneous activity in the precuneus, which was however positively linked to SPS. In other words, instead of accounting for the effect of dopamine-related genes on SPS, ReHo in the Pcc suppressed such an effect. After the suppressor effect was considered, the effect of dopamine-related genes on SPS became stronger. We speculate that this suppressor effect may be due to the precuneus’s role as part of the default mode network, whose activity is high at rest but low when engaged in tasks[54]. Further research is needed to replicate and explicate this suppressor effect.

In terms of the genetic mechanisms, little is known about the biochemical functions of the 10 SNPs associated with SPS. Several GWAS studies have included up to 8 of the 10 SNPs (except rs4929966 and rs16894446) (http://www.gwascentral.org/index), but they have not implicated these SNPs in the phenotypes investigated, either because their effects were too modest to survive the whole-genome correction or the phenotypes to which they contributed were not studied. Nevertheless, some of these SNPs or their genes were found in candidate gene studies to be significantly associated with sensitivity-related traits. Rs4929966 was associated with face emotion recognition [55], rs7131056 with social phobia and anxiety [56], and rs3842748 and rs7131056 with schizophrenia [57, 58], suggesting that these SNPs may influence sensitivity to emotion and social interaction. At the gene level, we previously summarized that these dopamine genes were often found to be associated with sensitivity-related traits [5]. For example, *TH* was related to essential hypertension; *DRD2* to childhood temperament, extraversion, and antisocial behavior; and neurotensin genes (*NLN*, *NTSR1*, *NTRS2*) to schizophrenia and memory consolidation. Other studies found that *DβH* was associated with cognitive impairment (Alzheimer's disease), working memory, and severity of psychotic symptoms [59–61], and *SLC6A3* with response inhibition [62]. We also found that *NTSR1* was associated with working memory [63]. Finally, there is a summary of associations between these dopamine-related genes and ADHD (http://adhd.psych.ac.cn/index.do). Taken together, these studies indicate that dopamine-related genes play many functions in the brain, some of which are subserved by brain regions such as the Pcc and lITG, and may influence SPS via cognitive and emotional mechanisms such as memory, attention, and emotional reactivity.

Several limitations of this study should be noted. First, the current study only revealed statistically significant associations among dopamine-related genes, brain function, and the sensitivity trait. Actual biological mechanisms involved need further investigation. Second, we only tested the contributions of dopamine-related genes to sensitivity and observed only a suppressor effect. Other neurotransmitter genes that are expressed within these brain regions (e.g. serotonin, GABA) [64] also need to be examined to see whether they showed mediation effects. Third, gene-environment interactions are likely to influence SPS, but they were not investigated in the current study. Given that SPS appears to be impacted by environmental factors [5, 65], especially by culture [15], a gene-environment interaction should be incorporated into the gene-brain-behavior model. Finally, we used intrinsic brain activation as the index of brain functions, genetic effects on other aspects of brain structure and functions such as structural volume and connectivity or task-evoked brain function also need to be explored.

## Supporting Information

S1 Table Subject data for gender, gene score, mean ReHo of mediation masks, and behavioral assessment. (DOC)
