# Supplementary material for: Regional Homogeneity of Resting-State Brain Activity Suppresses the Effect of Dopamine-Related Genes on Sensory Processing Sensitivity
Source: PLoS One. 2015 Aug 26;10(8):e0133143. doi: 10.1371/journal.pone.0133143 (PMC4550269; doi:10.1371/journal.pone.0133143)
Supplement: S1 Table — (DOC) [file pone.0133143.s001.doc]

***Supporting Information***

**Table. Subject data for gender, gene score, mean ReHo of mediation masks, and behavioral assessment**

| **ID** | **Gender^#^** | **genescore** | **ReHo-Pcc** | **ReHo-lITG** | **HSP** |
| --- | --- | --- | --- | --- | --- |
| SUB001 | 1 | 108.1701 | 2.2980 | 0.8251 | 98 |
| SUB002 | 1 | 110.1484 | 1.6755 | 0.5743 | 108 |
| SUB003 | 1 | 110.7958 | 2.2985 | 0.7467 | 62 |
| SUB004 | 2 | 111.4303 | 2.2260 | 0.6814 | 112 |
| SUB005 | 1 | 111.4303 | 2.1179 | 0.6333 | 114 |
| SUB006 | 2 | 111.4303 | 2.5258 | 0.7149 | 96 |
| SUB007 | 1 | 112.0777 | 2.3774 | 0.7382 | 113 |
| SUB008 | 2 | 112.2219 | 2.5907 | 0.4838 | 134 |
| SUB009 | 1 | 112.2219 | 2.1423 | 0.5982 | 97 |
| SUB010 | 2 | 112.6372 | 2.0252 | 0.6537 | 115 |
| SUB011 | 1 | 112.6372 | 2.0874 | 0.6961 | 103 |
| SUB012 | 2 | 112.6372 | 2.1890 | 0.7583 | 100 |
| SUB013 | 1 | 112.8693 | 2.2613 | 0.6639 | 130 |
| SUB014 | 2 | 113.3462 | 1.6366 | 1.0914 | 78 |
| SUB015 | 2 | 113.3462 | 2.0867 | 0.6242 | 140 |
| SUB016 | 2 | 113.6609 | 2.6613 | 1.0499 | 109 |
| SUB017 | 2 | 113.6609 | 2.0392 | 0.8852 | 94 |
| SUB018 | 1 | 114.1378 | 1.8585 | 0.7320 | 130 |
| SUB019 | 1 | 114.1378 | 2.8087 | 0.5656 | 123 |
| SUB020 | 1 | 114.2954 | 2.3788 | 0.8825 | 127 |
| SUB021 | 2 | 114.3069 | 2.3204 | 0.7108 | 112 |
| SUB022 | 2 | 114.7851 | 2.8462 | 0.8876 | 118 |
| SUB023 | 2 | 114.9428 | 2.1671 | 0.4368 | 112 |
| SUB024 | 2 | 115.087 | 2.3004 | 0.7147 | 113 |
| SUB025 | 1 | 115.087 | 2.1820 | 0.5158 | 136 |
| SUB026 | 2 | 115.087 | 2.1426 | 0.7654 | 98 |
| SUB027 | 2 | 115.087 | 1.7621 | 0.6398 | 126 |
| SUB028 | 2 | 115.1735 | 2.0829 | 0.6121 | 108 |
| SUB029 | 2 | 115.338 | 2.4515 | 0.7211 | 130 |
| SUB030 | 2 | 115.457 | 2.5513 | 0.7103 | 125 |
| SUB031 | 1 | 115.7344 | 1.7613 | 0.5080 | 128 |
| SUB032 | 1 | 115.7344 | 2.1407 | 0.7403 | 124 |
| SUB033 | 2 | 115.7344 | 2.2662 | 0.5309 | 156 |
| SUB034 | 1 | 115.8007 | 1.7735 | 0.5981 | 118 |
| SUB035 | 2 | 115.9651 | 1.8611 | 0.6356 | 125 |
| SUB036 | 2 | 115.9651 | 2.8985 | 0.7460 | 113 |
| SUB037 | 2 | 115.9853 | 1.9970 | 0.9049 | 144 |
| SUB038 | 2 | 115.9853 | 1.8601 | 0.5637 | 129 |
| SUB039 | 1 | 115.9853 | 2.3256 | 0.7810 | 122 |
| SUB040 | 2 | 116.0166 | 1.6470 | 0.6731 | 113 |
| SUB041 | 2 | 116.1295 | 1.7229 | 0.6833 | 100 |
| SUB042 | 1 | 116.1295 | 1.9415 | 0.6341 | 122 |
| SUB043 | 2 | 116.2113 | 1.7447 | 0.9107 | 95 |
| SUB044 | 1 | 116.2939 | 1.9782 | 0.8485 | 110 |
| SUB045 | 2 | 116.355 | 2.1830 | 0.8796 | 122 |
| SUB046 | 2 | 116.5259 | 2.1768 | 0.5804 | 86 |
| SUB047 | 2 | 116.6323 | 1.8500 | 0.7162 | 131 |
| SUB048 | 1 | 116.7567 | 2.1157 | 0.9521 | 110 |
| SUB049 | 2 | 116.7567 | 2.3654 | 0.6810 | 138 |
| SUB050 | 2 | 116.7567 | 2.1883 | 0.8493 | 107 |
| SUB051 | 2 | 116.8586 | 1.7518 | 0.6869 | 120 |
| SUB052 | 2 | 116.8586 | 2.2317 | 0.7257 | 129 |
| SUB053 | 1 | 116.8586 | 1.9314 | 0.5453 | 114 |
| SUB054 | 1 | 116.8586 | 2.1023 | 0.7446 | 89 |
| SUB055 | 2 | 116.8586 | 2.1432 | 0.4723 | 134 |
| SUB056 | 1 | 116.8832 | 1.9454 | 0.6658 | 103 |
| SUB057 | 2 | 116.9211 | 2.2734 | 0.8471 | 105 |
| SUB058 | 2 | 117.0029 | 2.5493 | 0.6108 | 141 |
| SUB059 | 1 | 117.0029 | 2.7960 | 0.6292 | 110 |
| SUB060 | 2 | 117.0029 | 2.5930 | 0.6259 | 114 |
| SUB061 | 2 | 117.0029 | 1.7632 | 0.6634 | 140 |
| SUB062 | 2 | 117.2538 | 1.8583 | 0.6285 | 122 |
| SUB063 | 2 | 117.2538 | 1.8659 | 1.0490 | 101 |
| SUB064 | 2 | 117.2796 | 2.4736 | 0.5291 | 114 |
| SUB065 | 2 | 117.4041 | 2.3666 | 0.8195 | 131 |
| SUB066 | 2 | 117.5685 | 3.0696 | 0.7871 | 109 |
| SUB067 | 1 | 117.6502 | 2.2881 | 0.6399 | 134 |
| SUB068 | 2 | 117.6502 | 2.5304 | 0.8218 | 133 |
| SUB069 | 1 | 117.6748 | 2.4075 | 0.8359 | 103 |
| SUB070 | 1 | 117.6748 | 2.0907 | 0.5713 | 116 |
| SUB071 | 2 | 117.7002 | 2.2609 | 0.5769 | 106 |
| SUB072 | 1 | 117.9257 | 1.6370 | 0.6671 | 106 |
| SUB073 | 1 | 118.1271 | 2.0004 | 0.8447 | 106 |
| SUB074 | 2 | 118.2097 | 2.2117 | 0.6764 | 130 |
| SUB075 | 2 | 118.2097 | 1.9924 | 0.8826 | 115 |
| SUB076 | 2 | 118.3221 | 2.4005 | 0.5237 | 131 |
| SUB077 | 1 | 118.5283 | 2.7157 | 0.8357 | 111 |
| SUB078 | 1 | 118.8302 | 1.9164 | 0.7380 | 95 |
| SUB079 | 2 | 118.8302 | 2.6077 | 0.6136 | 116 |
| SUB080 | 2 | 118.8817 | 1.8937 | 0.6591 | 144 |
| SUB081 | 2 | 118.9946 | 2.6058 | 0.5996 | 120 |
| SUB082 | 1 | 118.9946 | 2.3269 | 0.4876 | 126 |
| SUB083 | 1 | 119.0763 | 1.8663 | 0.9615 | 125 |
| SUB084 | 2 | 119.0763 | 2.1729 | 0.9402 | 102 |
| SUB085 | 1 | 119.0763 | 2.4943 | 0.9611 | 116 |
| SUB086 | 2 | 119.0763 | 2.7240 | 0.8611 | 114 |
| SUB087 | 2 | 119.0811 | 2.3546 | 0.9529 | 138 |
| SUB088 | 2 | 119.0811 | 1.9794 | 0.5184 | 129 |
| SUB089 | 2 | 119.4099 | 1.8592 | 0.5695 | 105 |
| SUB090 | 2 | 119.4165 | 2.1775 | 0.7261 | 125 |
| SUB091 | 2 | 119.4464 | 2.2163 | 0.6924 | 125 |
| SUB092 | 2 | 119.4698 | 2.4832 | 0.5743 | 103 |
| SUB093 | 2 | 119.471 | 2.3789 | 0.6301 | 113 |
| SUB094 | 2 | 119.6218 | 1.9139 | 0.4544 | 129 |
| SUB095 | 2 | 119.6218 | 2.3441 | 0.4933 | 108 |
| SUB096 | 2 | 119.6218 | 2.1252 | 0.6842 | 133 |
| SUB097 | 1 | 119.7237 | 2.0287 | 0.7313 | 100 |
| SUB098 | 2 | 119.7237 | 2.5896 | 0.8467 | 105 |
| SUB099 | 2 | 119.7237 | 1.9205 | 0.5580 | 129 |
| SUB100 | 1 | 119.7237 | 2.2558 | 0.7557 | 116 |
| SUB101 | 2 | 119.7237 | 2.9134 | 1.0946 | 98 |
| SUB102 | 1 | 119.7483 | 2.4500 | 0.7062 | 111 |
| SUB103 | 2 | 119.7483 | 2.2939 | 0.5645 | 93 |
| SUB104 | 1 | 119.7483 | 2.3567 | 0.5906 | 99 |
| SUB105 | 2 | 119.8679 | 1.6439 | 0.9081 | 101 |
| SUB106 | 2 | 119.8679 | 2.2219 | 0.5275 | 118 |
| SUB107 | 2 | 119.8679 | 1.7717 | 0.8591 | 125 |
| SUB108 | 2 | 119.8679 | 2.3422 | 0.7800 | 131 |
| SUB109 | 1 | 119.8679 | 1.6556 | 0.5779 | 130 |
| SUB110 | 2 | 119.9544 | 2.1891 | 0.7725 | 127 |
| SUB111 | 2 | 119.9544 | 2.0843 | 0.6036 | 112 |
| SUB112 | 1 | 120.1189 | 2.3332 | 0.9564 | 135 |
| SUB113 | 2 | 120.1189 | 2.3479 | 0.7784 | 121 |
| SUB114 | 2 | 120.1189 | 2.1772 | 0.5492 | 134 |
| SUB115 | 2 | 120.1189 | 2.0246 | 0.7838 | 103 |
| SUB116 | 2 | 120.2832 | 2.1111 | 0.5135 | 134 |
| SUB117 | 1 | 120.5153 | 2.0411 | 0.8186 | 116 |
| SUB118 | 1 | 120.5153 | 1.7877 | 0.9311 | 120 |
| SUB119 | 1 | 120.5399 | 2.5339 | 0.8765 | 118 |
| SUB120 | 1 | 120.5399 | 1.8369 | 0.6619 | 157 |
| SUB121 | 2 | 120.6018 | 1.8683 | 0.8149 | 130 |
| SUB122 | 2 | 120.6018 | 2.2445 | 0.6707 | 109 |
| SUB123 | 1 | 120.6018 | 2.0396 | 0.6971 | 120 |
| SUB124 | 1 | 120.6264 | 2.3180 | 0.6283 | 129 |
| SUB125 | 1 | 120.6264 | 2.0310 | 0.6233 | 133 |
| SUB126 | 2 | 120.746 | 1.9863 | 0.8499 | 133 |
| SUB127 | 2 | 120.746 | 2.0869 | 0.6768 | 138 |
| SUB128 | 1 | 120.746 | 2.2213 | 0.7337 | 120 |
| SUB129 | 2 | 120.7662 | 2.1296 | 0.8472 | 120 |
| SUB130 | 2 | 120.7908 | 1.5898 | 0.4503 | 148 |
| SUB131 | 2 | 120.9105 | 2.2802 | 0.7611 | 109 |
| SUB132 | 1 | 120.9551 | 1.9353 | 0.7660 | 108 |
| SUB133 | 2 | 120.9922 | 2.3665 | 0.5146 | 127 |
| SUB134 | 1 | 120.9922 | 2.8282 | 0.7043 | 106 |
| SUB135 | 2 | 120.9922 | 2.2820 | 0.6773 | 139 |
| SUB136 | 2 | 120.9922 | 1.8303 | 0.7372 | 132 |
| SUB137 | 1 | 121.0748 | 2.6101 | 0.6408 | 116 |
| SUB138 | 2 | 121.1613 | 1.7738 | 0.5988 | 116 |
| SUB139 | 2 | 121.3257 | 2.3736 | 0.7515 | 125 |
| SUB140 | 2 | 121.3886 | 2.1878 | 0.8144 | 108 |
| SUB141 | 2 | 121.3934 | 1.8948 | 0.5789 | 156 |
| SUB142 | 1 | 121.3934 | 1.9659 | 0.4801 | 123 |
| SUB143 | 2 | 121.418 | 2.4296 | 0.7783 | 111 |
| SUB144 | 1 | 121.6395 | 2.4443 | 0.6385 | 139 |
| SUB145 | 2 | 121.6395 | 2.0585 | 0.5913 | 124 |
| SUB146 | 2 | 121.6641 | 2.3607 | 0.7059 | 127 |
| SUB147 | 2 | 121.6641 | 2.1873 | 0.6417 | 155 |
| SUB148 | 1 | 121.6641 | 2.2274 | 0.5465 | 115 |
| SUB149 | 2 | 121.6641 | 2.0737 | 0.5163 | 144 |
| SUB150 | 2 | 121.6641 | 1.7128 | 0.7026 | 133 |
| SUB151 | 2 | 121.9462 | 1.9773 | 0.7103 | 138 |
| SUB152 | 2 | 122.3114 | 1.3950 | 0.9156 | 111 |
| SUB153 | 2 | 122.3361 | 2.3982 | 0.6901 | 121 |
| SUB154 | 2 | 122.3494 | 2.4629 | 0.6022 | 110 |
| SUB155 | 2 | 122.4311 | 2.5430 | 0.6182 | 113 |
| SUB156 | 2 | 122.4557 | 2.6101 | 0.5566 | 99 |
| SUB157 | 2 | 122.4557 | 2.5056 | 0.6083 | 109 |
| SUB158 | 1 | 122.4557 | 1.8953 | 0.6625 | 134 |
| SUB159 | 2 | 122.4557 | 1.6623 | 0.6722 | 119 |
| SUB160 | 2 | 122.4557 | 1.6053 | 0.9058 | 123 |
| SUB161 | 2 | 122.4557 | 2.5930 | 0.6325 | 107 |
| SUB162 | 1 | 122.4557 | 1.5259 | 0.7985 | 98 |
| SUB163 | 2 | 122.5734 | 1.7974 | 0.5093 | 134 |
| SUB164 | 2 | 122.5734 | 2.3366 | 0.7454 | 78 |
| SUB165 | 1 | 122.5754 | 1.8234 | 0.6023 | 158 |
| SUB166 | 2 | 122.7066 | 2.7332 | 0.6400 | 109 |
| SUB167 | 2 | 122.8195 | 2.1462 | 0.8699 | 127 |
| SUB168 | 2 | 122.871 | 2.8238 | 0.6423 | 112 |
| SUB169 | 2 | 123.103 | 2.1903 | 0.6458 | 122 |
| SUB170 | 2 | 123.103 | 2.1098 | 0.6229 | 147 |
| SUB171 | 1 | 123.2473 | 2.2211 | 0.7175 | 124 |
| SUB172 | 2 | 123.4535 | 2.2554 | 0.7714 | 114 |
| SUB173 | 1 | 123.4669 | 2.4480 | 0.7249 | 111 |
| SUB174 | 2 | 123.4669 | 2.2911 | 0.6269 | 130 |
| SUB175 | 2 | 123.5799 | 2.4696 | 0.4978 | 114 |
| SUB176 | 1 | 123.6111 | 2.2164 | 0.8730 | 130 |
| SUB177 | 1 | 123.6559 | 1.6510 | 0.8498 | 125 |
| SUB178 | 1 | 123.6976 | 1.7912 | 0.6918 | 126 |
| SUB179 | 2 | 123.7755 | 2.1384 | 0.7721 | 124 |
| SUB180 | 2 | 123.7755 | 2.2322 | 0.5956 | 134 |
| SUB181 | 2 | 123.8621 | 2.6438 | 0.5735 | 126 |
| SUB182 | 1 | 123.8621 | 2.4807 | 0.6340 | 134 |
| SUB183 | 1 | 123.8946 | 1.5406 | 0.5287 | 97 |
| SUB184 | 2 | 123.9193 | 2.5548 | 0.5360 | 110 |
| SUB185 | 1 | 124.0265 | 1.4517 | 0.7681 | 101 |
| SUB186 | 2 | 124.1908 | 2.2546 | 0.7967 | 115 |
| SUB187 | 2 | 124.2519 | 2.1734 | 0.7715 | 121 |
| SUB188 | 2 | 124.2585 | 1.6101 | 0.7231 | 124 |
| SUB189 | 2 | 124.2585 | 1.8096 | 0.5855 | 170 |
| SUB190 | 2 | 124.2585 | 2.6131 | 0.6359 | 115 |
| SUB191 | 2 | 124.2831 | 2.1393 | 0.8417 | 135 |
| SUB192 | 2 | 124.2831 | 2.6576 | 0.5857 | 126 |
| SUB193 | 2 | 124.4027 | 2.5929 | 0.5579 | 124 |
| SUB194 | 2 | 124.4027 | 2.8262 | 0.8401 | 122 |
| SUB195 | 1 | 124.4229 | 3.2764 | 0.6680 | 107 |
| SUB196 | 1 | 124.4475 | 2.1159 | 0.7061 | 131 |
| SUB197 | 2 | 124.4475 | 2.1320 | 0.5793 | 115 |
| SUB198 | 1 | 124.5292 | 2.0938 | 0.6501 | 124 |
| SUB199 | 2 | 124.5292 | 2.3828 | 0.6091 | 141 |
| SUB200 | 2 | 124.5292 | 2.3287 | 0.6766 | 123 |
| SUB201 | 2 | 124.5292 | 1.9080 | 0.9041 | 125 |
| SUB202 | 2 | 124.5671 | 2.1415 | 0.6415 | 132 |
| SUB203 | 2 | 124.6488 | 1.9662 | 0.6491 | 131 |
| SUB204 | 1 | 124.6488 | 1.0651 | 0.7687 | 110 |
| SUB205 | 1 | 124.6488 | 1.7925 | 0.6267 | 134 |
| SUB206 | 1 | 124.6536 | 2.1688 | 1.1143 | 119 |
| SUB207 | 2 | 124.6536 | 1.4963 | 0.5870 | 144 |
| SUB208 | 1 | 124.7568 | 2.3389 | 0.5953 | 109 |
| SUB209 | 2 | 125.0641 | 2.0328 | 0.4784 | 103 |
| SUB210 | 1 | 125.1765 | 2.1834 | 0.9344 | 137 |
| SUB211 | 2 | 125.2012 | 2.2150 | 0.4811 | 150 |
| SUB212 | 2 | 125.2962 | 1.8772 | 0.6820 | 135 |
| SUB213 | 1 | 125.3208 | 2.0742 | 0.5799 | 122 |
| SUB214 | 1 | 125.3208 | 2.1015 | 0.6835 | 117 |
| SUB215 | 2 | 125.3208 | 2.4726 | 0.6485 | 105 |
| SUB216 | 2 | 125.3208 | 1.8850 | 0.7427 | 127 |
| SUB217 | 2 | 125.3208 | 2.4944 | 0.6816 | 124 |
| SUB218 | 2 | 125.4073 | 2.4372 | 0.5622 | 113 |
| SUB219 | 2 | 125.4073 | 1.6352 | 0.5900 | 91 |
| SUB220 | 2 | 125.4452 | 1.8701 | 0.7232 | 133 |
| SUB221 | 1 | 125.6097 | 2.1916 | 0.6995 | 136 |
| SUB222 | 2 | 125.6135 | 2.6307 | 0.5818 | 122 |
| SUB223 | 2 | 125.6914 | 2.5814 | 0.5689 | 127 |
| SUB224 | 1 | 125.9422 | 2.0740 | 0.6268 | 120 |
| SUB225 | 2 | 125.9681 | 1.9644 | 0.6284 | 109 |
| SUB226 | 1 | 126.1989 | 2.3283 | 0.7367 | 124 |
| SUB227 | 2 | 126.3387 | 2.0273 | 0.7242 | 130 |
| SUB228 | 1 | 126.3633 | 2.1563 | 0.6073 | 122 |
| SUB229 | 2 | 126.3633 | 2.1100 | 0.5848 | 133 |
| SUB230 | 2 | 126.3633 | 2.3934 | 0.6415 | 127 |
| SUB231 | 2 | 126.445 | 2.2686 | 0.6028 | 130 |
| SUB232 | 1 | 126.445 | 1.8941 | 0.6229 | 132 |
| SUB233 | 2 | 126.5277 | 2.8590 | 0.7397 | 161 |
| SUB234 | 2 | 126.5277 | 2.6088 | 0.8456 | 127 |
| SUB235 | 2 | 126.6473 | 2.3286 | 0.6765 | 117 |
| SUB236 | 2 | 126.7351 | 2.2363 | 0.7359 | 138 |
| SUB237 | 2 | 126.7446 | 1.8682 | 0.6205 | 133 |
| SUB238 | 2 | 126.8916 | 2.8621 | 0.8417 | 104 |
| SUB239 | 1 | 127.0924 | 2.0514 | 0.7240 | 103 |
| SUB240 | 1 | 127.117 | 2.1506 | 0.6098 | 126 |
| SUB241 | 2 | 127.3231 | 2.2960 | 0.6572 | 124 |
| SUB242 | 2 | 127.4875 | 2.3111 | 0.5997 | 122 |
| SUB243 | 1 | 127.4875 | 1.9464 | 0.5957 | 115 |
| SUB244 | 2 | 127.6004 | 2.1977 | 0.7370 | 116 |
| SUB245 | 2 | 127.9041 | 2.5252 | 0.5541 | 138 |
| SUB246 | 2 | 127.9086 | 2.4561 | 0.4909 | 153 |
| SUB247 | 2 | 127.9086 | 2.4672 | 0.5576 | 133 |
| SUB248 | 2 | 128.0988 | 2.1720 | 0.5765 | 135 |
| SUB249 | 2 | 128.2724 | 2.1728 | 0.6144 | 141 |
| SUB250 | 2 | 128.4368 | 2.5811 | 0.7216 | 135 |
| SUB251 | 1 | 128.4435 | 2.1955 | 0.6477 | 152 |
| SUB252 | 2 | 128.4543 | 2.5518 | 0.4922 | 129 |
| SUB253 | 2 | 129.0394 | 2.2495 | 0.5049 | 126 |
| SUB254 | 1 | 129.0394 | 2.2337 | 0.6085 | 132 |
| SUB255 | 2 | 129.0394 | 1.7513 | 0.6438 | 107 |
| SUB256 | 2 | 129.064 | 2.5457 | 0.5472 | 113 |
| SUB257 | 2 | 129.064 | 2.2007 | 0.7388 | 139 |
| SUB258 | 2 | 129.2038 | 1.8636 | 0.6647 | 131 |
| SUB259 | 1 | 129.2284 | 2.6215 | 0.6712 | 140 |
| SUB260 | 1 | 129.3101 | 1.7810 | 0.6626 | 148 |
| SUB261 | 2 | 129.3101 | 2.2957 | 0.7350 | 136 |
| SUB262 | 2 | 129.3481 | 2.4490 | 0.6441 | 126 |
| SUB263 | 2 | 129.8244 | 2.5018 | 0.7490 | 122 |
| SUB264 | 1 | 129.8244 | 2.9941 | 0.8186 | 130 |
| SUB265 | 2 | 129.831 | 2.3379 | 0.6905 | 141 |
| SUB266 | 2 | 129.9821 | 1.7688 | 0.7201 | 119 |
| SUB267 | 1 | 129.9821 | 1.9060 | 0.6505 | 136 |
| SUB268 | 2 | 130.0994 | 1.9055 | 0.5245 | 125 |
| SUB269 | 2 | 130.1017 | 2.1617 | 0.5759 | 120 |
| SUB270 | 2 | 130.749 | 1.8861 | 0.6644 | 117 |
| SUB271 | 2 | 130.7737 | 2.0250 | 0.5581 | 139 |
| SUB272 | 2 | 130.9798 | 2.0373 | 0.7821 | 125 |
| SUB273 | 2 | 131.0174 | 2.7607 | 0.5955 | 143 |
| SUB274 | 2 | 131.232 | 2.4187 | 0.7530 | 117 |
| SUB275 | 2 | 131.3086 | 2.4657 | 0.4219 | 139 |
| SUB276 | 1 | 131.4781 | 1.4841 | 0.7905 | 128 |
| SUB277 | 2 | 131.5081 | 1.8142 | 0.6028 | 114 |
| SUB278 | 2 | 131.5255 | 2.3275 | 0.6809 | 144 |
| SUB279 | 1 | 131.8979 | 1.8187 | 0.9296 | 146 |
| SUB280 | 2 | 132.0046 | 2.0372 | 0.5936 | 125 |
| SUB281 | 1 | 132.111 | 1.9750 | 0.4906 | 120 |
| SUB282 | 2 | 133.0533 | 2.8747 | 0.6817 | 128 |
| SUB283 | 2 | 133.2106 | 2.3831 | 0.9099 | 133 |
| SUB284 | 1 | 133.2352 | 1.9443 | 0.7619 | 128 |
| SUB285 | 2 | 133.2352 | 2.0709 | 0.4977 | 139 |
| SUB286 | 2 | 133.8449 | 1.8286 | 0.8659 | 132 |
| SUB287 | 2 | 133.8449 | 2.0793 | 0.6036 | 142 |
| SUB288 | 2 | 133.8449 | 2.2471 | 0.4762 | 167 |
| SUB289 | 1 | 133.8449 | 1.8051 | 0.6531 | 92 |
| SUB290 | 2 | 134.6365 | 2.2372 | 0.6699 | 118 |
| SUB291 | 2 | 134.6741 | 2.1769 | 0.6185 | 157 |
| SUB292 | 2 | 136.1003 | 2.3238 | 0.5998 | 103 |
| SUB293 | 2 | 136.1003 | 2.9686 | 0.6879 | 125 |
| SUB294 | 1 | 136.7476 | 1.9955 | 0.6480 | 120 |
| SUB295 | 1 | 139.1715 | 2.1132 | 0.8218 | 147 |
| SUB296 | 1 | 139.2542 | 2.8199 | 0.5870 | 159 |
| SUB297 | 2 | 139.9631 | 1.9565 | 0.6576 | 153 |
| SUB298 | 2 | 140.8812 | 2.2105 | 0.5516 | 148 |

Note: ReHo = regional homogeneity, Pcc = precuneus, lITG = left inferior temporal gyrus;

^#^ Gender: 1 = male, 2 = female.
